# An Optimization Method for Enterprise Resource Integration Based on Improved Particle Swarm Optimization

**DOI:** 10.1155/2022/6928989

**Published:** 2022-05-31

**Authors:** Aifang Guo, Lina Zhu, Lingjie Chang

**Affiliations:** Department of Economy and Trade, Changzhi Vocational and Technical College, Changzhi 046000, China

## Abstract

An enterprise's development and growth are inextricably linked to rational and efficient resource integration and optimization. This study focuses on the reorganization and integration of industrial elements inside the firm from the standpoint of resource integration. The ideal resource integration strategy is investigated by integrating the industrial parts of a certain enterprise in order to increase the efficiency of project completion and lower enterprise expenses. The enterprise's internal material and human resources are limited, but it is frequently necessary to execute numerous activities at the same time, and each activity must meet multiple goals. This research investigates how to properly integrate and schedule resources while attaining different goals. This research proposes using an enhanced particle swarm optimization technique (IPSO) to combine firms' internal resources. In order to address the issue of uneven particle dispersion caused by random population initialization, IPSO incorporates chaos theory into particle population initialization. The logistic mapping sequence generates a huge number of particles, and the particles with the highest quality are chosen for initialization. This can increase particle quality, allowing particles to be spread equally during setup. In the late stage, the classic particle swarm optimization algorithm (PSO) has a slow convergence rate, causing the algorithm to readily slip into a local optimal solution. This research proposes a dynamic inertia weight update approach based on fitness value. In the later stages of the algorithm, this strategy can improve the convergence speed and quality of the global optimal solution, allowing the particles to do a global search and eventually identify the population's ideal solution. Furthermore, IPSO creates a fitness function depending on task completion time. IPSO is used to test the performance of an enterprise's resource integration case. Experiments show that the method utilized can swiftly locate the ideal solution, complete the integration, and optimization of enterprise resources in the shortest job completion time, and for the least amount of money.

## 1. Introduction

Rapid advancements in science and technology have resulted in rapid economic growth. Economic development also poses difficulties for the management and operation of various businesses. To stand firm in today's rapidly changing society, businesses must not only have a long-term development plan on the outside but also a reasonable and efficient resource integration mechanism on the inside. The ability of an enterprise to screen, acquire, and combine various heterogeneous resources in the process of operation, including both the macrolevel that the enterprise obtains resources from the external environment where it is located, and the internal the microlevel of resource allocation. Some academics divide enterprise resource integration capabilities into macrolevel categories based on this. The macrolevel enterprise resource integration capabilities include enterprises' ability to reconstruct operating rules and predict corporate strategies and risks in advance, whereas the microlevel enterprise resource integration capabilities include enterprises' ability to allocate, stimulate, and combine various existing resources. Existing research has examined the factors that may influence the development of enterprise resource integration capabilities in various contexts. According to the resource-based viewpoint, an enterprise is a collection of resources that can be obtained in a variety of ways. Resources are an important factor in an enterprise's long-term development. If a company wants to gain a competitive advantage and establish a market presence, it must invest in valuable resources and management skills. Unrepeatable strategic management resources that can assist companies in successfully managing their businesses and obtaining additional compensation. At the moment, many scholars support the theory of resource-based view, and most studies are aimed at the impact of a specific type of enterprise's own resources on enterprise performance. Many of the results of the experimental analyses are consistent with resource-based theory. The uniqueness and nonreplicability of resources can play a role in the operation of businesses, forming competitive advantages and determining enterprise performance. Each enterprise's management resources are unique, and they have varying degrees of importance at each stage of the enterprise's operation. It can be defined as the various human, financial, and material resources that business managers manage in their daily operations. The good operation of the enterprise's resources can achieve the management goals of the enterprise.

Enterprise managers with varying management capabilities deal with their management resources in various ways. Reasonable resource allocation and utilization can result in satisfactory operational results. According to reference [1] the materials owned by the enterprise cannot directly create performance and obtain benefits for the enterprise. Professional resource integration talents, on the other hand, can integrate the enterprise's static resources and generate benefits for the enterprise. Differences in management resources will result in differences in resource allocation efficiency, resulting in differences in corporate performance. According to reference [2], each company has distinct management resources and management employees with different talents. As a result, each business will develop its own management style. This type of management disparity resulting from resource differences will have an impact on the company's performance. Firms with a wealth of management resources outperform their competitors. External mergers and acquisitions by businesses, according to reference [3], might boost a company's ability to manage resources swiftly. The outside offers fresh resources and management strategies to the company, which will boost the company's ability to adapt to changes in the external environment while also promoting its growth. Rich management resources, according to reference [4], can assist businesses optimize and alter their organizational structure to react to changing external market conditions. At the same time, businesses can create long-term plans and alter their management practices as needed. Reference [5] demonstrates that firms can strengthen their market position by fully utilizing management resources. To summarize, resources play an essential role in the development of enterprises, and management resources play a positive function in enhancing firm performance, according to resource-based theory. The better the managerial resources, the better the company's performance.

Enterprise resource management optimization's ultimate goal is to maximize the company's benefits. To optimize earnings, different companies have different criteria. It mostly entails optimizing earnings and reducing the time it takes to execute tasks. To do this, the enterprise's material and human resources must be integrated and dispatched in a reasonable manner. Enterprise resource integration and scheduling are NP-complete problems [6, 7]. Particle swarm algorithms [8–10], ant colony algorithms [11–13], simulated annealing algorithms [14–16], and genetic algorithms [17–19] are currently available that can solve NP-complete problems. The PSO algorithm has the advantages of fast convergence speed, easy operation, and the algorithm is convenient for global optimization, but it also has disadvantages such as easy to fall into local optimum and low search accuracy. Aiming at these problems, this paper proposes an IPSO algorithm, and applies it to the instance of enterprise resource integration and scheduling. In order to verify the performance of the method used in this paper, the experimental results obtained through simulation experiments show that the IPSO algorithm has a good effect on solving the problem of enterprise resource integration.

## 2. Information Construction of Enterprise Resource Management

### 2.1. Enterprise Resource Management Platform

**Figure 1:**
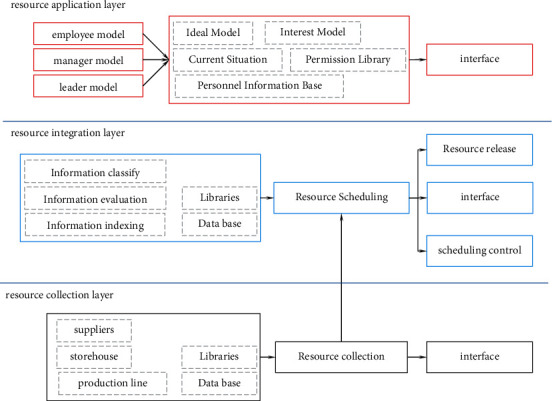
Architecture of enterprise resource integration platform.

Business resources are not only the most significant aspect of an enterprise's internal environment, but they are also the prerequisites for enterprise resource integration. Enterprise management is now completely reliant on computers, and the advancement of information technology has expedited the reform of internal management and resource management in businesses. As a result, a series of information systems focused on the integration and deployment of enterprise resources have emerged. The system can help with resource integration and give a platform for all employees to quickly acquire tasks and resources.  Figure 1 depicts the enterprise resource integration optimization platform's design structure.

Figure 1 depicts a three-tier architecture for an enterprise resource integration platform. The acquisition layer gathers the data that the system requires. The resource integration layer supports various resource release and interface negotiation models, as well as evaluating and classifying the released data before storing it in various databases. To achieve intelligent business resource integration, the resource application layer executes application processing on diverse enterprise information resources.

### 2.2. Optimization of Enterprise Resource Integration

**Figure 2:**
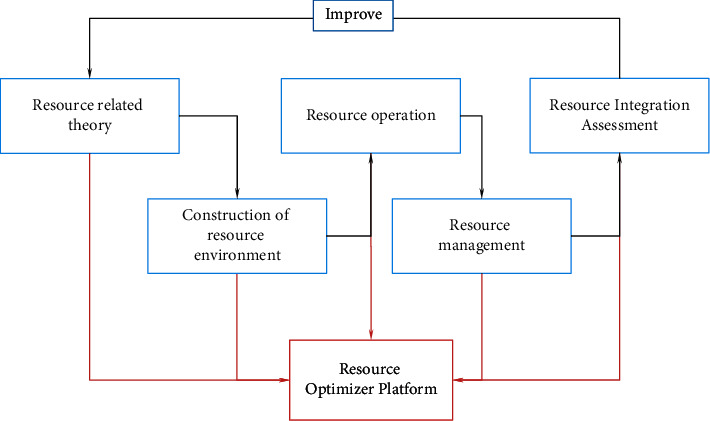
Optimization of enterprise resource integration.

Optimizing enterprise resource integration mainly starts from four aspects: resource creation, resource use, integration, and effect evaluation. The relationship and interaction of these four aspects are shown in Figure 2.

**Figure 3:**
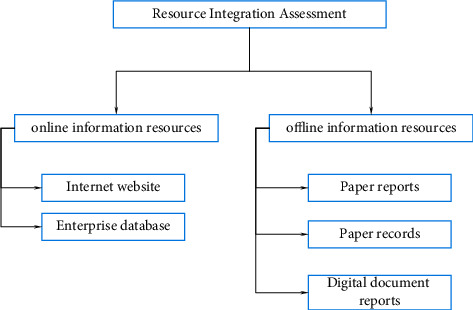
Enterprise resource integration assessment process.

1. Theory is an important basis for guiding actual production. Constructivist theory mainly emphasizes the initiative and situational nature of resource integration. Resource integration is not a stimulus to passively receive information, but to actively process external information according to the corporate background. When the resource content is related to the needs of the enterprise, the enterprise should propose appropriate resource integration and allocation strategies to deal with various tasks. The resource integration dependence theory mainly emphasizes that the survival of the entire enterprise organization needs to absorb resources from the surrounding environment, and the interaction between the organization and the control resources in other environments is carried out.
2. Resource creation. Resource creation should take enterprise managers as the main body and employees' needs as the fundamental starting point, so as to encourage employees, managers, and leaders to work together. Build a resource environment dominated by service resources to ensure the reliability of resources, thereby providing guarantees for sustainable development of resources.
3. Independent integration management includes user information maintenance, resource information maintenance, and integration strategy. User information maintenance requires periodic updates and confidentiality of information. Enterprises need to set confidential information goals, formulate information integration plans, determine integration progress, and select integration resources to achieve self-maintenance. Resource information maintenance is to store and update resource information. Integrate and optimize resources based on integration strategies.
4. Evaluation is the guarantee for resource integration. With the goal of resource integration and optimization, the design evaluation process is shown in Figure 3. Figure 3 shows that evaluation is an important indicator to measure the rational integration of resources and is the driving force behind the integration of resources and information within an enterprise. To evaluate based on the integration of platform resources, it is not only necessary to analyze each link but also to evaluate the effect of self-integration of employee information in a timely manner.

## 3. Enterprise Resource Integration and Optimization of IPSO Algorithm

### 3.1. Introduction to PSO

Suppose a population *X* is searched in a *D*-dimensional space, and each population consists of *H* particles. Each particle in the population can be represented as a solution to the problem. *X*_*i*_(*t*) to represent the position of the *i*-th particle in the *t*-th iteration of the population. *V*_*i*_(*t*) is the velocity of the particle at the *t*-th iteration in the population. *S*_*i*_ represents the self-optimal solution of the *i-*th particle, and *S*_best_ represents the optimal solution of the entire particle population. The mathematical expression formula of each variable is as follows:

(1) $$\begin{array}{c} X\left(t\right)=\left\{X_{1}\left(t\right),X_{2}\left(t\right),\ldots ,X_{H}\left(t\right)\right\},\\ X_{i}\left(t\right)=\left(x_{i1}\left(t\right),x_{i2}\left(t\right)\ldots ,x_{iD}\left(t\right)\right),\\ V_{i}\left(t\right)=\left(v_{i1}\left(t\right),v_{i2}\left(t\right)\ldots v_{iD}\left(t\right)\right),\\ S_{i}=\left(S_{i1,}S_{i2,}\ldots ,S_{iD}\right),\\ S_{\text{best}}=\left(S_{1,}S_{2,}\ldots ,S_{D}\right). \end{array}$$

PSO updates the state of the particle through the particle's velocity and position; equation (2) is the velocity update expression, and equation (3) is the position update expression:

(2) $$\begin{array}{c} v_{i}\left(t+1\right)=wv\left(t\right)+e_{1}g_{1}\left(S_{i}\left(t\right)-x_{i}\left(t\right)\right)+e_{2}g_{2}\left(S_{\text{best}}-x_{i}\left(t\right)\right), \end{array}$$

(3) $$\begin{array}{c} x_{i}\left(t+1\right)=x_{i}\left(t\right)+v_{i}\left(t+1\right), \end{array}$$

where *w* is the inertia weight coefficient, which is used to determine how much velocity is retained after the last iteration; *e*1, *e*2 are the learning factors of the algorithm. Usually, both parameters are set to 2. *g*1, *g*2 are random numbers whose values are between [0, 1]; t is the number of iterations variable; and *T* is the maximum number of iterations. [*X*_min_, *X*_max_] is the value range of position, and [*V*_min_, *V*_max_] is the value range of velocity. The fitness value function *f* is used to judge the quality of particles. The size of the fitness function value indicates the pros and cons of the problem solution. After the particles are iterated, equation (4) is the update formula of the individual optimal value. When the maximum number of iterations is reached, the algorithm stops iterating:

(4) $$\begin{array}{c} S_{i}=\left\{\begin{array}{cc} X_{i}\left(t\right), & f\left(X_{i}\left(t\right)\right)>f\left(S_{i}\right),\\ S_{i}, & f\left(X_{i}\left(t\right)\right)\leq f\left(S_{i}\right). \end{array}\right. \end{array}$$

**Figure 4:**
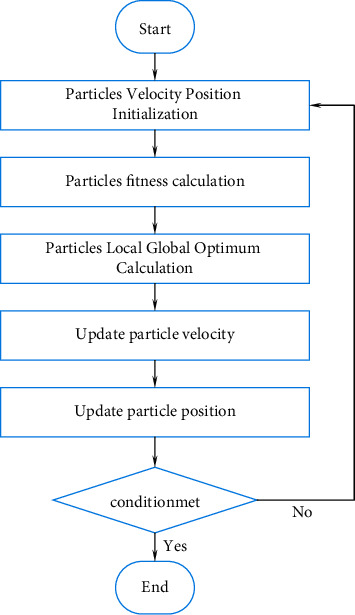
PSO flow chart.

The flow of the PSO algorithm is shown in Figure 4.

The specific implementation steps of PSO are as follows:

Step 1: Initialize the particle swarm. Given *t*=0, bring the value of *t* into the particle velocity formula and position formula, and determine the initial position of the particle as *X*_*i*_(0) and the initial velocity as *V*_*i*_(0). Set all parameter values related to the algorithm, such as *H*, *D*, *T*, *w*, *e*1, *e*2, *g*1, *g*2.
Step 2: Calculate the fitness value of each particle.
Step 3: Set the fitness value of the current position of the particle as the optimal solution *S*_*i*_ of the particle itself. Set the optimal solution in the initial population as *S*_best._
Step 4: Update the particles.
  ① Update the position of the particle according to equation (3), and update the velocity of the particle according to equation (2).
  ② Judging the velocity and position of the current particle within a given range.
  ③ Calculate the fitness value of each particle, and then update *S*_*i*_ according to equation (4). Compare the fitness value of the individual optimal position and the group optimal position to update *S*_best._
Step 5: Judge whether the end condition is met, if the end condition is met, then step 6 is executed. Otherwise, the number of iterations is increased by 1, and step 4 is executed.
Step 6: Output *S*_best_, and the algorithm ends.

### 3.2. IPSO Algorithm

Due to the slow convergence speed of the traditional PSO late stage, this will result in the inability to perform global search in the particle late stage. Aiming at this problem, this paper improves the update method of inertia weight value, which can improve the convergence ability and search ability of the algorithm. In addition, since the population initialization of PSO is random, this will inevitably lead to a part of the particles far away from the optimal solution and affect the quality of the particles. Therefore, in this paper, chaos theory is added to the population initialization process to improve the particle quality and optimization ability.

Choosing an appropriate inertia weight *w* is the key to improving the convergence and optimization capabilities of PSO. The size of the inertia weight value determines the optimization ability of the algorithm. When the inertia weight value is small, the local optimization ability of the algorithm is better. When the inertia weight value is large, the global optimization ability of the algorithm is better. However, in the traditional PSO, the inertia weight value is set to a fixed value; so many studies have proposed various improvement strategies for the inertia weight to make the optimization ability of the algorithm the best. Among the many improvement strategies, the improvement ideas of the following strategies are very good. The strategy can update the inertia weight value according to the number of iterations. The ability of particle local optimization and global optimization is balanced by dynamically adjusting the *w* value. The mathematical expression for this strategy is as follows:

(5) $$\begin{array}{c} w=w_{\max}-\frac{w_{\max}-w_{\min}}{T}\times t, \end{array}$$

where *w*_max_ represents the maximum inertia weight value, and *w*_min_ represents the minimum inertia weight value. In the early stage of the algorithm search, because the number of iterations is relatively small, the value of *w* will be large, which is convenient for particles to search globally. In the later stage of the algorithm search, as the value of *t* increases, the value of *w* will become smaller, which makes it easier for the particle to find the local optimal solution.

Inspired by the above strategies, this paper proposes a new method to update inertia weights. This method also dynamically updates the inertia weight based on the size of the fitness value. *f*_avg_ represents the average fitness value of the population. *f*_*i*_ represents the fitness value of the particle currently being iterated, *i*=1,2,3,…*H*, *H* is the population size. The inertia weight value ranges from 0.4 to 0.9. The inertia weight expression used in this paper is as follows:

(6) $$\begin{array}{c} v_{i}\left(t+1\right)=wv_{i}\left(t\right)+e_{1}g_{1}\left(S_{\text{best}}-x_{i}\left(t\right)\right),\\ s.Tw=\left\{\begin{array}{cc} w_{\min}+\frac{f_{\text{avg}}-f_{i}}{f_{\text{avg}}}, & f_{i}\leq f_{\text{avg}},\\ w_{\max}-\frac{f_{\text{i}}-f_{\text{avg}}}{f_{\text{avg}}}, & f_{i}\geq f_{\text{avg}}. \end{array}\right. \end{array}$$

Chaos theory has different chaotic mapping methods, this paper adopts Logistic mapping. The principle of logistic mapping is the regression equation. Logistic maps are well ergodic and the particles are evenly distributed over their range. Its regression equation is as follows:

(7) $$\begin{array}{c} y_{k+1,j}=4y_{k,j}\left(1-y_{k,j}\right)y_{k,j}\in \left(0,1\right), \end{array}$$

where *k* is the number of iterations, and *y*_*kj*_ is the population sequence. This article will use chaos theory to initialize the population. PSO uses the chaotic sequence to produce a large number of particles during initialization and selects the particles with better quality as the initial particles of the population. This ensures that the particles are uniformly distributed in the solution space and the quality of the particles can be guaranteed.

(1) The steps to initialize using the chaotic sequence are as follows:
  Step 1. Randomly generate a number *y*_0,0_ in [0, 1].
  Step 2. Bring *y*_0,0_ into equation (8) for iteration to generate the sequence *y*_*kj*_.
  Step 3. Repeat step 2 until *k*=2^*∗*^*H*;
  Step 4. Map the generated sequence to the solution space of the population according to equation (8) so that 2^*∗*^*H* particles can be obtained.
    (8) $$\begin{array}{c} x_{k,j}=a+y_{k,j}\left(b-a\right), \end{array}$$
  where *a*=*X*_min_, *b*=*X*_max_.
  Step 5. Select the optimal *H* particles from the generated 2^*∗*^*H* particles as the initial population.
(2) The implementation steps of particle swarm optimization based on chaos theory are as follows:
  Step 1. Population initialization based on chaos theory. Set the initial iteration number *t* to 0, and other parameters in the algorithm.
  Step 2. Calculate the fitness value of the selected *H* particles with good quality.
  Step 3. Set the fitness value of the current position of the particle as the optimal solution *S*_*i*_ of the particle itself. Set the optimal solution in the initial population as Sbest.
  Step 4.Update the particle's position and velocity:
  ①  Update the velocity and position of the particle according to the relevant update formula.
  ②  Determine whether the current particle velocity and position are beyond the feasible range.
  ③  Update *S*_*i*_ and Sbest according to equation (4).
  Step 5. Determine whether the end condition is reached. If the end condition is reached, execute step 6. If not, increase the number of iterations by 1, and execute step 4.
  Step 6. Output *S*_best_, and the algorithm ends.

## 4. Construction and Solution of Resource Integration Model Based on IPSO

### 4.1. Model Building

A single project has a clear project goal in the project planning stage and is divided into several tasks through work breakdown, and these tasks are all serving the project goal. Generally speaking, the scope of project objectives includes three dimensions: cost, time, and quality. In the multiproject management environment, the implementation of all projects of the enterprise serves the enterprise strategy. Project resource scheduling meets the project's time requirements for different resources as much as possible and strives to keep the completion time of all projects as short as possible. At the same time, such scheduling will inevitably involve organizational resources, so how to improve the utilization efficiency of organizational resources becomes a problem. In addition, the project leader will also pay attention to cost and quality issues. On the one hand, he hopes to reduce the project budget and improve the project income, and on the other hand, the quality should be as high as possible.

Under the premise of limited total resource supply, the multiproject resource scheduling problem can be regarded as the superposition of resource scheduling problems in multiple tiny time intervals. Therefore, it is possible to take a certain period of time during the execution of the project for analysis. Suppose the multiproject resource scheduling problem involves *m* mutually independent projects and *n* resource supply types. Within the time interval Δ*T*, there are *e* processes in progress in project *i*. Where the quantity of the *k*^th^ resource required by the *j*^th^ process is denoted as *z*_*i*,*j*,*k*_, the quantity of the obtained resource is denoted as *n*_*i*,*j*,*k*_, and the total supply of this type of resource is *Z*_*k*_. Once the construction period delay in project *i* causes losses, the total cost of delaying the project per unit time is recorded as *s*_*i*_. Δ*t*_*i*_ represents the engineering delay time of project *i* within the time interval Δ*T*. With the goal of minimizing project losses due to insufficient resource supply, the objective function is established, as follows:

(9) $$\begin{array}{c} Z=\min\sum_{i=1}^{q}\Delta t_{i}s_{i}, \end{array}$$

where *s*_*i*_ is a known quantity, which can be known from the contract; Δ*t*_*i*_ is an unknown quantity, which is determined by the quantity *z*_*i*,*j*,*k*_ of resources obtained by the process *j* performed by the project *i* in the time period Δ*T*. Under the condition that the total supply of various resources is limited, *n*_*i*,*j*,*k*_ should meet the restrictive conditions:

(10) $$\begin{array}{c} \sum_{i=1}^{q}\sum_{j=1}^{q}n_{ijk}\leq Z_{k},k=1,2,\ldots ,w. \end{array}$$

In the time period Δ*T*, the number of resources required by the critical path process in project *i* is *z*_*izk*_, and the number of resources obtained is lick. Let *t*_*iz*_ denote the planned working time of the critical path, *t*_*iz*_=Δ*T*, and *t*′_*iz*_ denote its actual working time. From the principle that the total consumption of each resource in the same process remains unchanged, that is, *z*_*izk*_*t*_*iz*_=*n*_*izk*_*t*_*iz*_′, *t*_*iz*_′=*z*_*izk*_*t*_*iz*_/*n*_*izk*_,  *k*=1,2,…, *w*. Therefore, the delay time of the critical path of project *i* can be expressed and known as

(11) $$\begin{array}{c} \Delta t_{i}=\Delta t_{iz}=\frac{z_{izk}-n_{izk}}{n_{izk}}. \end{array}$$

In the same way, for any process on the noncritical path in project *i*, mark the number of required resources as *r*_*isk*_, and the number of obtained resources as *n*_*isk*_, within the time period Δ*T*, the planned working time of this path is *t*_*if*_, and the time difference is *t*_*is*_. The delay time is expressed as follows:

(12) $$\begin{array}{c} \Delta t_{is}=\max\left\{\frac{t_{is}\left(z_{\text{isk}}-n_{isk}\right)}{n_{\text{isk}}}\right\}. \end{array}$$

In order to ensure that the critical path of project *i* does not change, the difference between the delay time of the noncritical path and the time difference owned by the process should not exceed the delay time of the key process, namely,

(13) $$\begin{array}{c} \Delta t_{is}-T_{is}\leq \Delta t_{i}. \end{array}$$

In the actual project implementation, the construction of any process may require multiple resources at the same time, and a certain proportional relationship must be satisfied between different resources to coordinate and promote the process forward. In resource scheduling, under any circumstances, for the ongoing process *j* in project *i*, the supply quantity of resources and the demand quantity should satisfy the following proportional relationship:

(14) $$\begin{array}{c} n_{ijk}=\eta_{ij}z_{ijk},\;k=1,2,\ldots ,w,\;0<\eta_{ij}\leq 1, \end{array}$$

where *η*_*ij*_ represents the resource allocation coefficient related to each process *j* under construction within the time interval Δ*T*.

To sum up, substituting equations (7) and (11) into equations (9), (10) and (13) respectively, we get

(15) $$\begin{array}{c} Z=\min\left[\Delta T\sum_{i=1}^{q}s_{i}\left(\frac{1}{\eta_{iz}}-1\right)\right], \end{array}$$

(16) $$\begin{array}{c} \sum_{i=1}^{q}\sum_{j=1}^{q}\eta_{ij}z_{ijk}\leq Z_{k},\;k=1,2,\ldots ,w, \end{array}$$

(17) $$\begin{array}{c} t_{is}\eta_{iz}\leq \Delta T\eta_{is}. \end{array}$$

Equations (15)–(17) constitute a mathematical model of enterprise resource scheduling in a multiproject environment with limited resources.

### 4.2. Model Solution

The IPSO algorithm is proposed to be used for enterprise resource integration and scheduling. In order to verify the effectiveness of the proposed algorithm for enterprise resource integration scheduling, this paper introduces the method into a practical case. A company has a total of four projects to be completed within a certain period of time, and there are three types of resources needed and available. Each project has a different number of processes. Every project has strict delivery time requirements. The loss cost *M*_*i*_ (*i* = P1,P2,P3,P4) of each item per delay unit time is 1000 for *M*_P1_, *M*_P2_ = 800, *M*_P3_ = 1500, *M*_P4_ = 1800.  Table 1 is a breakdown of the resource requirements of the enterprise to complete each project, where Δ*T* is set to 5.

Substitute the data shown in Table 1 into the resource integration model shown in equation (15), and the obtained formula is as follows:

(18) $$\begin{array}{c} \min z=5*1000\left(\frac{1}{x_{3}}-1\right)+5*800\left(\frac{1}{x_{5}}-1\right)+5*1500\left(\frac{1}{x_{6}}-1\right)+5*1800\left(\frac{1}{x_{10}}-1\right),\\ s.t.18x_{1}+15x_{2}+10x_{3}+29x_{4}+17x_{5}+13x_{6}+21x_{7}+16x_{8}+23x_{9}+18x_{10}\leq 150\\ 25x_{1}+27x_{2}+16x_{3}+18x_{4}+23x_{5}+26x_{6}+10x_{7}+18x_{8}+15x_{9}+11x_{10}\leq 190\\ 9x_{1}+16x_{2}+35x_{3}+18x_{4}+12x_{5}+11x_{6}+14x_{7}+23x_{8}+14x_{9}+8x_{10}\leq 150\\ 4x_{3}\leq 5x_{1}\\ 2x_{3}\leq 5x_{2}\\ 3.5x_{5}\leq 5x_{4}\\ 4x_{6}\leq 5x_{7}\\ 4.5x_{10}\leq 5x_{8}\\ 3x_{10}\leq 5x_{9}\\ 0<x_{i}<1, \end{array}$$

where *x*_1_, *x*_2_, *x*_3_ represents the resource allocation coefficient of process P11, P12, P13; *x*_4_, *x*_5_ represents the allocation coefficient of process P21, P22, *x*_6_, *x*_7_ represents the resource allocation coefficient of process P31, P32; *x*_8_, *x*_9_, *x*_10_ represents the resource allocation coefficient of process P41, P42, P43.

For the above mathematical programming model, the standard particle swarm optimization algorithm and the new PSO optimization algorithm proposed in this paper are used to solve the problem, respectively. The parameter settings of the algorithm are evolution times *T* = 600, population size *D* = 300, and parameter *e*_1_ = 1.532, *e*_2_ = 1.541, the error *ε* = 0.0001.

**Figure 5:**
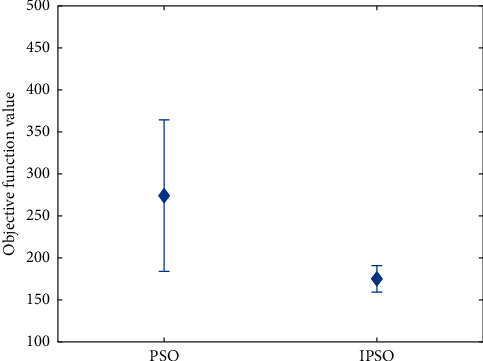
Comparison of project loss costs obtained by different algorithms.

In addition, Figure 5 shows the simulation results of the two algorithms. It can be seen from the figure that the average value of the project loss cost obtained by the standard particle swarm optimization method is larger than the result obtained by the new particle swarm optimization algorithm. According to the discrete degree of the objective function, the simulation results of the new particle swarm optimization algorithm are more concentrated, while the simulation results of the standard PSO [20] are more scattered.

After the above comparison and analysis of the simulation results of the two algorithms, it is concluded that compared with the standard particle swarm optimization algorithm, the improved particle swarm optimization algorithm in this paper has higher stability and better convergence.

According to the resource allocation coefficients obtained by the PSO and IPSO algorithms, the allocation of each resource among different projects is calculated, and the resource allocation of the two algorithms is obtained as shown in Tables 2 and 3.

Observing the data in Table 2, it can be seen that the allocation of resource R1 based on PSO is 136.17, the allocation of resource R2 is 152.63, and the allocation of resource R3 is 121.07. There is a large gap between the calculated allocation and supply of each resource. This shows that the allocation of resources is not reasonable. The delay times for the three operations of project P1 are 1.489, 3.119, and 2.765, respectively. The planned time differences corresponding to these three processes are 0, 2, and 2.5, respectively. The delay time calculated based on PSO is larger than the planned time difference. This shows that resource integration based on PSO is not very effective. The delay times for the two processes of project P2 are 2.120 and 2.221 respectively. The planned time differences corresponding to these two processes are 3 and 2, respectively. It can be seen that the process P21 can be completed within the set time difference, but the process P22 cannot be completed within the set time difference. The delay times for the two processes of project P3 are 1.765 and 0.815 respectively. The planned time differences corresponding to these two processes are 1.5 and 2, respectively. It can be seen that the process P32 can be completed within the set time difference, but the process P31 cannot be completed within the set time difference. The delay times for the three operations of project P4 are 2.642, 0 and 2.812, respectively. The planned time differences corresponding to these three processes are 3, 1, and 3, respectively. From the comparison data, it can be seen that these three processes can be completed within the set time difference. To sum up, in the resource integration scheme based on the PSO algorithm, except for P4, which can be completed according to the set time difference, other projects cannot be completed within the set time difference.

Observing the data in Table 3, it can be seen that the allocation of resource R1 based on IPSO is 159.74, the allocation of resource R2 is 175.89, and the allocation of resource R3 is 137.41. The calculated allocation of each resource is very close to the available Supply amount. This shows that the allocation of resources is more reasonable. In the results calculated based on IPSO, the delay times for the three processes of project P1 are 1.622, 2.120 and 3.235, respectively. The planned time differences corresponding to these three processes are 0, 2, and 2.5, respectively. The delay time calculated based on IPSO is larger than the planned time difference. The delay times for the two processes of project P2 are 1.522 and 1.767, respectively. The planned time differences corresponding to these two processes are 3 and 2, respectively. It can be seen that both steps P21 and P22 can be completed within the set time difference. The delay time for the 2 processes of project P3 is 0.521,0 respectively. The planned time differences corresponding to these two processes are 1.5 and 2, respectively. It can be seen that both steps P31 and P32 can be completed within the set time difference. The delay times for the three processes of project P4 are 2.764, 1.656, and 2.701, respectively. The planned time differences corresponding to these three processes are 3, 1, and 3, respectively. From the comparison data, it can be seen that this process P41 and P43 can be completed within the set time difference, and P42 cannot be completed within the set time difference. To sum up, in the resource integration scheme based on the PSO algorithm, except for P2 and P3, which can be completed according to the set time difference, other projects cannot be completed within the set time difference.

**Figure 6:**
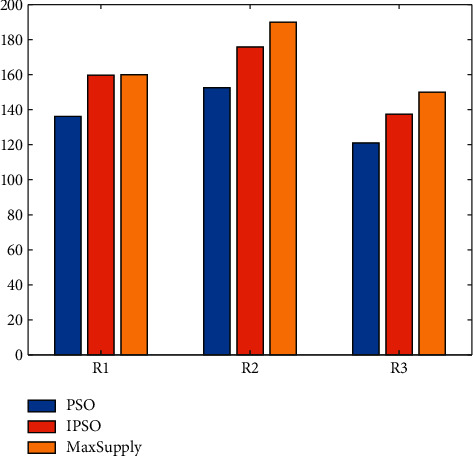
Comparison of resource allocation of different algorithms.

In order to compare the resource integration and allocation schemes obtained by the two methods more intuitively, screen out the optimal resource allocation scheme, and give a resource allocation comparison chart, as shown in Figure 6. In the figure, MaxSupply represents the amount of resources that the enterprise can supply. From the data in the figure, it can be seen that the allocation of the three resources based on IPSO is the closest to the maximum supply. However, there is still a big gap between the distribution scheme based on PSO and the maximum supply.

## 5. Conclusion

In the process of continuous operation and development of enterprises, there will definitely be some problems in the use and deployment of internal resources. In order to optimize the integration and allocation of enterprise resources, this paper proposes to use an optimization algorithm to allocate resources. Its purpose is to efficiently complete the delivery of each project within the contracted construction period. Based on the traditional PSO algorithm, this paper proposes an IPSO algorithm. Firstly, to solve the problem of uneven particle distribution caused by random initialization of the population, the algorithm adds chaos theory to the initialization of particle population. It uses the Logistic mapping sequence to generate a large number of particles, and selects the particles with better quality for initialization, which improves the quality of the particles and enables the particles to be evenly distributed during initialization. Secondly, the late convergence speed of particle swarm optimization algorithm is slow, which makes it easy to fall into the local optimal solution. Aiming at this problem, a dynamic inertia weight update method based on fitness value is designed. This can improve the convergence speed in the later stage of the algorithm, improve the quality of the global optimal solution, and enable the particles to perform a global search and finally find the optimal solution of the population. Finally, a fitness function based on task completion time is designed, and the IPSO algorithm is applied to the integration and optimization of internal resources of the enterprise. Compared with the standard particle swarm optimization algorithm, the IPSO algorithm has higher stability and better convergence. In the experimental part, a set of optimal solutions are selected to solve the resource allocation of each project process in the experimental example. The results show that the IPSO algorithm can produce better results for this kind of resource allocation problem. It can not only allocate the limited enterprise resources reasonably, but also minimize the total project loss caused by the delay of the construction period. The optimization results show that the IPSO algorithm proposed in this paper can effectively integrate and schedule the internal resources of the enterprise and improve the operation efficiency of the enterprise.

## Figures and Tables

**Table 1:** Details of enterprise resource requirements.

Project	Process	Required time	Time difference	Resource R1	Resource R2	Resource R2

P1	P11	4	0	18	25	9
	P12	2	2	15	27	16
	P13	5	2.5	10	16	35

P2	P21	3.5	3	29	18	18
	P22	5	2	17	23	12

P3	P31	5	1.5	13	26	11
	P32	4	2	21	10	14

P4	P41	4.5	3	16	18	23
	P42	3	1	23	15	14
	P43	5	3	18	22	8

Resource requirements				**180**	**200**	**160**
Max supplies				150	190	150

**Table 2:** Details of the actual allocation of enterprise resources based on PSO.

Project	Process	Required time	Time difference	Resource R1	Resource R2	Resource R2	Partition coefficient	Delay value
P1	P11	4	0	13.33	18.51	6.66	0.7403	1.489
	P12	2	2	12.38	22.28	13.20	0.8251	3.119
	P13	5	2.5	6.56	10.49	22.95	0.6557	2.765

P2	P21	3.5	3	13.20	8.20	8.20	0.4553	2.120
	P22	5	2	9.29	12.57	6.56	0.5465	2.221

P3	P31	5	1.5	13.00	26.00	11.00	1	1.765
	P32	4	2	19.50	9.29	13.00	0.9287	0.815

P4	P41	4.5	3	14.08	15.84	20.24	0.8798	2.642
	P42	3	1	23.00	15.00	14.00	1	0
	P43	5	3	11.84	14.47	5.26	0.6576	2.812

Original resource requirement				180	200	160		
Actual allocation of each resource				136.17	152.63	121.07		
Max supplies				160	190	150		

**Table 3:** Details of the actual allocation of enterprise resources based on IPSO.

Project	Process	Required time	Time difference	Resource R1	Resource R2	Resource R2	Partition coefficient	Delay value
P1	P11	4	0	17.21	23.90	8.60	0.9561	1.622
	P12	2	2	15.00	27.00	16.00	1	2.120
	P13	5	2.5	7.76	12.41	27.14	0.7755	3.235

P2	P21	3.5	3	24.54	15.23	15.23	0.8462	1.522
	P22	5	2	12.48	16.88	8.81	0.7341	1.767

P3	P31	5	1.5	11.33	22.66	9.59	0.8715	0.521
	P32	4	2	21.00	10.00	14.00	1	0

P4	P41	4.5	3	12.06	13.57	17.34	0.7539	2.764
	P42	3	1	22.20	14.48	13.51	0.9652	1.656
	P43	5	3	16.16	19.75	7.18	0.8978	2.701

Original resource requirement				180	200	160		
Actual allocation of each resource				159.74	175.89	137.41		
Max supplies				160	190	150		

## Data Availability

The labeled data set used to support the findings of this study is available from the corresponding author upon request.
